# Expression of α_v_ Integrin in Feline Injection-Site Sarcoma (FISS): Preliminary Investigations

**DOI:** 10.3390/ani14243588

**Published:** 2024-12-12

**Authors:** Andrea Cappelleri, Eleonora Brambilla, Lavinia E. Chiti, Alessia Trapletti, Gaia B. M. Bianchi, Mauro Di Giancamillo, Valeria Grieco, Chiara Giudice

**Affiliations:** 1 Department of Veterinary Medicine and Animal Sciences, University of Milan, 26900 Lodi, Italy; andrea.cappelleri@unimi.it (A.C.); eleonora.brambilla@unimi.it (E.B.); trapletti99@gmail.com (A.T.); gaiabeatrice.bianchi@unimi.it (G.B.M.B.); mauro.digiancamillo@unimi.it (M.D.G.); valeria.grieco@unimi.it (V.G.); 2 Clinic for Small Animal Surgery, Department for Small Animals, Vetsuisse Faculty, University of Zurich, 8057 Zurich, Switzerland; laviniaelena.chiti@uzh.ch

**Keywords:** FISS, integrin, immunohistochemistry, fibrosarcoma, pleomorphic sarcoma, injection-site granuloma

## Abstract

Feline injection-site sarcomas (FISSs) are malignant skin tumors of mesenchymal origin arising at local post-vaccination (or injection) sites. In recent years, a fluorescence imaging technique based on probes targeting α_v_β_3_ integrin has been effectively applied for the surgical complete resection of the tumor. In our study, we investigated the utility of a commercially available anti-α_v_ integrin polyclonal antibody for the histopathological evaluation of FISS’s surgical excision margins. We collected 10 formalin-fixed paraffin-embedded (FFPE) feline excisional biopsies with a histopathological diagnosis of FISS (7 fibrosarcomas and 3 pleomorphic sarcomas) and wide margin tissue, along with one subcutaneous injection-site granuloma and 6 osteosarcomas. Samples were processed for histology, and slides were stained for IHC with the anti-α_v_ integrin antibody. Immunostained slides were evaluated for the cellular localization and intensity of the staining in different neoplastic and non-neoplastic cell populations. Neoplastic and non-neoplastic spindle cells had cytoplasmic positivity in all fibrosarcomas. Inflammatory cells, including macrophages of the injection-site granuloma, were negative. Multinucleated giant cells in the pleomorphic sarcomas had an intense membranous positivity. Although the anti-α_v_ integrin antibody was ineffective for the histopathological evaluation of surgical excision margins, the membranous localization of α_v_ integrin in multinucleated giant cells of pleomorphic sarcomas suggests that it plays a role in the oncogenesis of this FISS variant.

## Simple Summary

Feline injection-site sarcomas (FISSs) are malignant skin tumors of mesenchymal origin arising at local post-vaccination (or injection) sites due to the neoplastic transformation of fibroblasts and myofibroblasts. In recent years, a fluorescence imaging technique based on probes targeting α_v_β_3_ integrin was effectively applied as optical guidance in the surgical complete resection of the tumor. In our study, we investigated the utility of a commercially available antibody directed against α_v_ integrin for the histopathological evaluation of FISS’s surgical excision margins. Although the antibody did not allow us to mark neoplastic cells over reactive ones in the biopsies, an interesting membranous positivity was found in multinucleated giant cells of the pleomorphic sarcoma variant of FISS, suggesting a specific role for α_v_ integrin in the oncogenesis of this subtype of FISS.

## 1. Introduction

Feline injection-site sarcomas (FISSs) are malignant skin tumors of mesenchymal origin whose etiopathogenesis is still poorly understood. Currently, the most widely accepted hypothesis to explain their onset is a local post-vaccination (or injection) inflammatory process that leads to the neoplastic transformation of fibroblasts and myofibroblasts [1,2,3,4,5,6,7]. In fact, FISS most frequently appears at the site of vaccinations, but it also appears at the sites of the injection of foreign material, trauma, or microchip implantation [2,3,8,9]. The mechanism by which the inflammatory reaction causes tumor formation is not fully understood, but the overexpression of growth factors and oncogene activation have been demonstrated in cats with FISS and are suspected to play a role in tumor development [6]. Most of these tumors are fibrosarcomas, but rhabdomyosarcomas, myxosarcomas, chondrosarcomas, osteosarcomas, and pleomorphic sarcomas have also been reported [2,6,10,11]. FISSs are characterized by a variable, often long latency period, rapid growth, and high local aggressiveness, despite a low metastatic rate [2,3,10].

The first-line therapy for FISS is curative-intent surgical excision. Depending on the primary site and the involvement of adjacent bone structures, radical excision, including spinous vertebral process amputation, partial or total scapulectomy, rib resection, or limb amputation, may be necessary [1,3]. Some important limitations of surgical interventions are the low tumor accessibility, difficult visual tumor delineation, micro-metastasis detection, and damage to vital structures [12]. There are a few clinical intra-operative imaging techniques that are currently used in both human and veterinary medicine to aid the distinction between tumor and normal tissue during the surgical procedure, thus maximizing the chances of complete resection, such as magnetic resonance imaging (MRI), ultrasound (US), computed tomography (CT), and X-ray fluoroscopy [12]. Fluorescence imaging recently emerged as a valuable method to guide the surgeon during tumor resection. This is an optical technique that implies the use of molecular probes labeled with near-infrared (NIR) fluorophores, allowing the real-time intra-operative assessment of tumor borders [12,13]. In recent years, an NIR imaging-based method that exploits a fluorescent RGD-based probe targeting α_v_β_3_ integrin has been applied as optical guidance in surgery on spontaneous feline fibrosarcoma [12]. This method relies on the transmembrane protein receptor α_v_β_3_ integrin’s high affinity for the RGD motif, found in several extracellular matrix (ECM) proteins, such as fibronectin, vitronectin, and laminin [14,15,16,17,18]. The reason why α_v_β_3_ integrin represents a promising molecule to target tumor tissues is that it is highly expressed on the surface of several tumor cell types and angiogenic endothelial cells but is expressed at very low levels on quiescent endothelial cells and other tissues [12,17,18]. In fact, integrins are involved in various cancer stages such as malignant transformation, tumor growth and progression, invasion, and metastasis [17]. In light of its unique expression in tumor tissues, α_v_β_3_ integrin has been evaluated as a marker of tumor extent, aiding in surgical interventions for FISS with clean margins [12].

Notwithstanding the enhanced precision of surgical techniques, the histopathology of the surgical margins is still imperative in assessing the complete excision of the tumor [1,5]. However, histopathology is not without pitfalls. In instances where the neoplasm is clearly present within margin tissues, as well as when it is absent, the histopathological interpretation of margin status is relatively straightforward. However, a degree of uncertainty remains in cases where the margins are affected by the presence and proliferation of hyperplastic fibroblasts, which are frequently associated with a variable degree of lymphocytic, plasmacytic, and macrophage infiltration and newly formed vessels. Distinguishing between neoplastic and reactive fibroblasts within the septa of connective tissue of the margins is perhaps the main challenge for the pathologist.

In light of the in vivo intra-surgical application of integrin as a tumor marker, we hypothesized that integrin could serve as a specific label for neoplastic cells in fixed tissues, thereby offering a valuable tool for the assessment of the status of FISS margins.

Therefore, in the present study, we investigated the immunohistochemical expression of α_v_ integrin in feline formalin-fixed paraffin-embedded (FFPE) biopsies of margin tissue in cases with a histological diagnosis of FISS, to verify the differential expression of this marker between neoplastic and non-neoplastic portions.

## 2. Materials and Methods

A total of 10 FFPE feline excisional biopsies with a histopathological diagnosis of FISS (7 fibrosarcomas and 3 pleomorphic sarcomas) and with available margin tissues were retrieved from the archive of the Unit of Pathology of the Department of Veterinary Medicine and Animal Sciences (DIVAS—Lodi, Università degli Studi di Milano). Neoplasms were considered consistent with FISS when they were located at an anatomical site used for vaccine or drug administration, had a clinical history of drug administration, and had histological features that have been reported to be typical of FISS, such as the presence of necrosis and the inflammatory infiltration of mononuclear cells [6,19].

Samples were collected during routine diagnostic procedures, and the use of residual amounts of tissues and/or samples for research purposes was approved by the ethical committee (OPBA_60_2022). Biopsies were previously trimmed according to the 3D histology technique [7] and routinely processed for histology, as previously described. Briefly, for the 3D histology technique, a strip of 3–4 mm thickness was obtained, following the entire perimeter of the surgical specimen, and further subdivided into smaller segments that could fit into plastic cassettes for histological preparations. Each margin specimen was laid flat in the cassette with the external side down to obtain a histological slide containing the outermost margin of the surgical specimen. A complete longitudinal section throughout the neoplastic mass, including all the closest superficial, cutaneous, and deep muscular layers, was also obtained.

In 5 cases, the histological margins were recorded as being free of tumor infiltration; in one case, the tumor extended to the cut borders, and in 4, the histological interpretation of the margin status was doubtful.

One subcutaneous injection-site granuloma and 6 osteosarcomas (OSAs) were also included for comparison. Given that OSA can be a subtype of FISS, endosteal OSAs, and tumors located at an anatomical site other than those commonly utilized for vaccine or drug administration (e.g., distal limbs or orbit), were selected. Four-micrometer thick sections were obtained from the paraffin blocks and stained with hematoxylin and eosin (HE). Immunohistochemistry (IHC) for α_v_ integrin was performed with the avidin–biotin complex (ABC) method. A rabbit polyclonal anti-α_v_ integrin primary antibody (AB1930, Merck, Darmstadt, Germany) was selected for the study since it was previously used in the feline species on FFPE samples [20].

For IHC, tissue sections were dewaxed in xylene and hydrated through graded alcohols. Heat-induced epitope retrieval was performed using citrate buffer, with pH 6.5, at boiling temperature in a pressure cooker for 18 min. Endogenous peroxidase was blocked with 3% H_2_O_2_ for 20 min at room temperature. Non-specific protein binding was prevented with 10% normal goat serum for 30 min at 4 °C. Sections were then incubated for 18 h at 4 °C with the rabbit polyclonal anti-α_v_ integrin primary antibody. Various serial dilutions were tested for the primary antibody, and a dilution of 1:1000 was selected for providing optimal immunostaining results. Sections were eventually incubated for 20 min at room temperature with the BOND Polymer Refine Red Detection System Reagent 1 (Leica Biosystems, Newcastle Upon Tyne, UK), followed by incubation for 20 min at room temperature with the BOND Polymer Refine Red Detection System Reagent 2 (Leica Biosystems, Newcastle Upon Tyne, UK). The reaction was visualized with the NovaRED substrate kit (VectorLabs, Torrance, CA, USA). Sections were counterstained with Mayer’s hematoxylin and mounted with Micromount (Diapath, Martinengo, Italy). Endothelial cells were used as internal positive controls. Negative controls were carried out on seriate sections by replacing the primary antibody with rabbit IgG (sc2027, Santa Cruz, Dallas, TX, USA).

HE-stained slides were evaluated using a light microscope by 2 board-certified pathologists (A. C. and C. G.) to confirm the diagnosis. Particular attention was given to the re-evaluation of surgical margins. Immunostained slides were evaluated for the cellular localization (membranous, cytoplasmic) and the intensity of the staining (−, no staining; +, mild positive staining; ++, moderate positive staining +++, intense positive staining) in the following cell populations: endothelial cells, spindle neoplastic cells, mononucleated round neoplastic cells, multinucleated giant neoplastic cells, spindle cells in the margins of the surgical excision, and inflammatory cells (lymphocytes, plasma cells and macrophages). For the interpretation of immunostained slides, the whole histopathological section was evaluated at high magnification, avoiding areas of necrosis and artifacts.

## 3. Results

The cats affected by FISS included 6 females and 3 males; the sex of one subject was unknown. At the time of surgery, patients were between 10 and 21 years old (mean 13.5, median 13). Regarding the location of the FISS, 4 were interscapular, 3 were in the thoracic wall subcutis, one was on the flank, one was on the thigh, and one case was on the trunk, but the exact location was not recorded.

The histopathological diagnosis of FISS was confirmed using HE-stained slides in all cases. FISSs were consistently characterized by variably sized areas of necrosis and, in some cases, by large necrotic–cystic areas. Inflammatory cell infiltrates were seen in all samples, with lymphocytes being the predominant cell type, and a small number of macrophages and plasma cells were also present.

In 2 cases, intense granulomatous panniculitis was present in the subcutaneous tissue surrounding the neoplasia, and in one of these cases, macrophages containing a globular grayish intracytoplasmic material were also present.

Seven cases were classified as the fibrosarcoma subtype. Tumors were composed of multiple and often coalescing nodules that infiltrated and expanded the subcutis downward from the panniculus carnosus muscle. Areas of necrosis of variable extent were present in all cases, and in 3 cases were associated with necrotic–cystic areas. Neoplastic cells were plump and spindle-shaped and were arranged in interwoven bundles immersed in a variable amount of eosinophilic fibrillar ground substance (collagen), which, in one case, multifocally, had a loose mucinous appearance. The number of mitoses ranged from 2 to 62 (mean 24, median 14) in 2.37 mm^2^.

Three cases were classified as the pleomorphic sarcoma subtype, characterized by spindle cells admixed with numerous mononucleated round cells and multinucleated giant cells. The spindle-shaped cells had a faintly basophilic cytoplasm with indistinct cell borders, round-to-fusiform nuclei with finely stippled chromatin, and multiple small nucleoli. The giant cells were characterized by multiple, round-to-oval nuclei (up to 60) with dense chromatin and inconspicuous nucleoli. Anisocytosis and anisokaryosis were marked in all cases, and 10 to 59 mitoses (mean 29.6, median 20) in 2.37 mm^2^ were observed.

Surgical margins were classified as free from tumor infiltration in 5 FISSs (3 fibrosarcomas and 2 pleomorphic sarcomas), with doubtful tumor infiltration in 4 FISSs (fibrosarcomas), and infiltrated in one FISS (pleomorphic sarcoma).

Free margins consisted of a mixture of mature adipose tissue interspersed with septa of dense fibrovascular connective tissue and skeletal muscle. The fibrous tissue contained mature, well-differentiated fibrocytes with slender, dense nuclei and sparse fusiform eosinophilic cytoplasm. Sparse fibroblasts were occasionally present in the fibrous septa. Multifocally, lymphocytes and plasma cells were occasionally present in small perivascular aggregates.

Doubtful margins also consisted of a mixture of connective fibrovascular tissue, adipose tissue, and skeletal muscle. Fibrous septa contained both fibrocytes and fibroblasts. The latter were large spindle cells, single and scattered or organized in short loose bundles and characterized by oval nuclei and moderate amounts of eosinophilic cytoplasm. Mild anisocytosis and anisokaryosis were common, while mitotic figures were extremely rare. Frequently, lymphocytes, plasma cells, and, more rarely, macrophages were associated with the hyperplastic fibroblasts. In the sections examined, this branching proliferative fibrous tissue was not clearly continuous with the neoplasia, although continuity in another plane could not be excluded.

Infiltrated margins were invaded by bundles of neoplastic cells, continuous with the main lesion and characterized by histopathological features overlapping with those of the tumor described above.

The cats with OSA included 5 females and one male, between 8 and 13 years old (mean 9.7, median 9). The OSAs were composed of moderately pleomorphic, spindle-shaped fibroblast-like cells and plump, oval cells with basophilic cytoplasm and eccentric, hyperchromatic nuclei. Ribbons or islands of osteoid, small bone trabeculae, and round, multinucleated (osteoclast-like) cells were also present.

The granulomatous lesion developed at the site of injection on the right shoulder of a 3-year-old female cat, 1 month after vaccination. Histologically, multifocal to coalescing, variably sized, grossly nodular lesions substituted the normal architecture of the subcutaneous adipose tissue. Nodules were partially demarcated by an incomplete rim of intensely vascularized immature fibrous connective tissue, rich in fibroblasts. Large centers of colliquative necrosis, surrounded by a rim of epithelioid or foamy macrophages interspersed with eosinophilic granulocytes and a smaller number of lymphocytes and plasma cells, were frequently present. Alternatively, the nodules had a solid center composed of dense aggregates of epithelioid macrophages with abundant finely granular weakly eosinophilic cytoplasm. Scattered aggregates of lymphocytes, at various stages of maturation and organized in follicle-like structures, were present at the periphery of the lesions. Multiple small solid nodules composed of epithelioid macrophages were scattered throughout the fibro-adipose tissue surrounding the main lesion, associated with mild perifocal fibroblast proliferation.

Regarding IHC results, in all cases, endothelial cells (internal positive control) were intensely positive within the neoplasia, in mesenchymal tissues surrounding the neoplasia, and in the margins of the samples.

**Figure 1:**
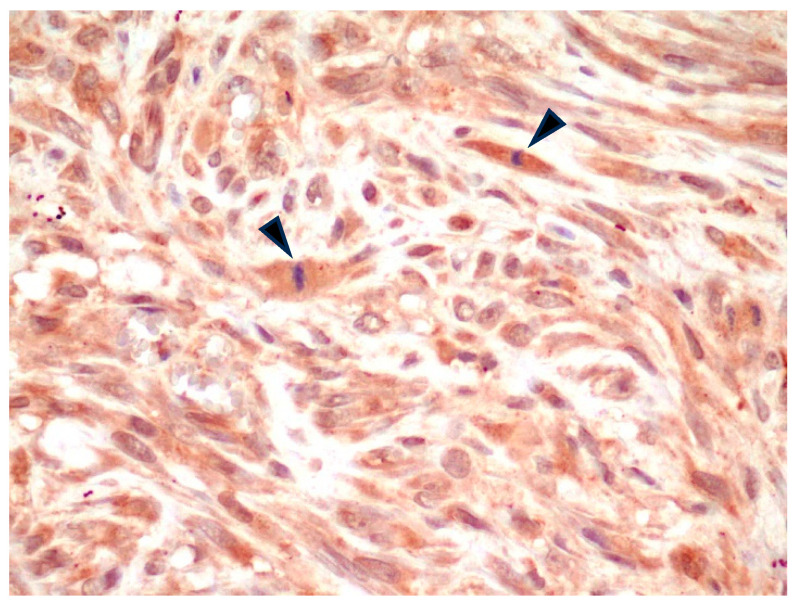
FISS, fibrosarcoma. Moderate cytoplasmic positivity for α_v_ integrin. Note the presence of at least 2 mitotic figures in the field (arrowheads). IHC for α_v_ integrin, 400×.

All FISS were positive for α_v_ integrin (Table 1) with a variable pattern of cell staining. Specifically, in 7/7 fibrosarcomas, spindle neoplastic cells were characterized by mild-to-moderate cytoplasmic positive immunostaining (Figure 1). Nuclear positivity was occasionally noted in the same cells and interpreted as non-specific.

**Figure 2:**
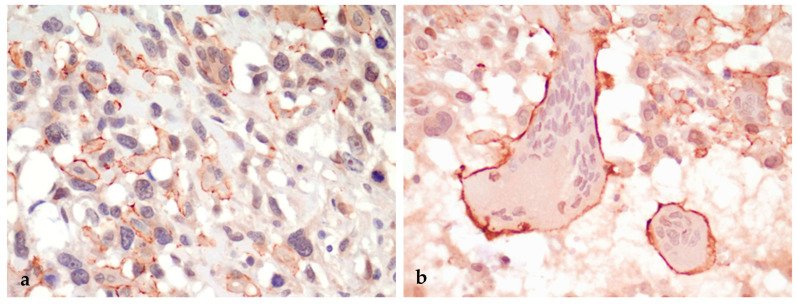
FISS, pleomorphic sarcoma. (**a**) Spindle neoplastic cells are mostly negative for α_v_ integrin, whereas round and multinucleated giant neoplastic cells display intense membranous labeling. (**b**) Detail of 2 voluminous multinucleated giant neoplastic cells displaying strong membranous labeling. IHC for α_v_ integrin, 400×.

In all (3/3) pleomorphic sarcomas, spindle neoplastic cells had negative to mildly positive cytoplasmic immunostaining, while mononucleated round neoplastic cells and multinucleated giant neoplastic cells were characterized by intense membranous immunostaining (Figure 2a,b).

Osteosarcomas were characterized by mild-to-moderate cytoplasmic positivity in both spindle-shaped (fibroblast-like) and oval (osteoblast) neoplastic cells. Multinucleated giant cells (interpreted as osteoclasts) had mild-to-moderate intracytoplasmic staining and inconsistent, faint membranous positive staining (2/6 cases).

In all the examined cases, inflammatory cells (lymphocytes, plasma cells, and, when present, macrophages) within the neoplasia, in the margin tissues, and composing the areas of panniculitis were consistently negative.

**Figure 3:**
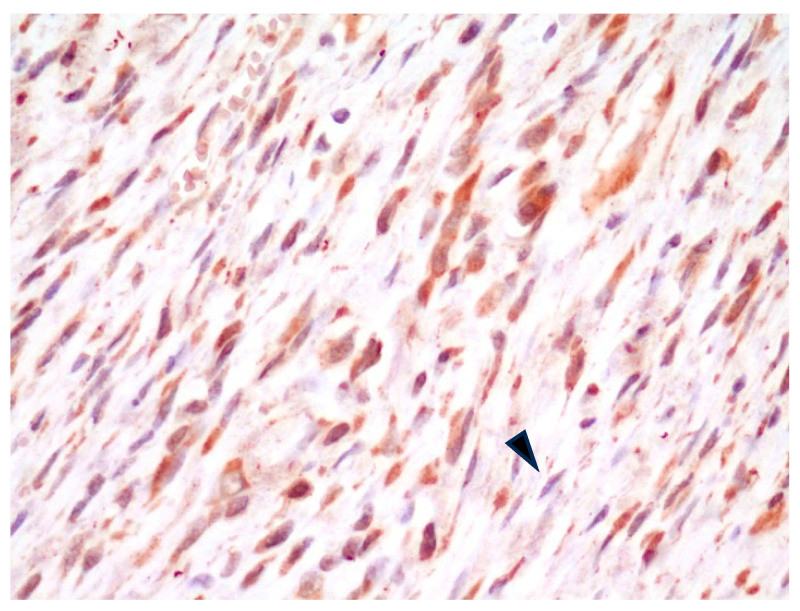
FISS, margin of fibrosarcoma. Fibroblasts have diffuse, mild-to-moderate cytoplasmic positivity for α_v_ integrin, whereas fibrocytes are mildly stained or negative (arrowhead). IHC for α_v_ integrin, 400×.

In all FISS cases, in the tissue composing the surgical margins, regardless of the histopathological interpretation of the margin status, spindle cells with plump nuclei and moderate cytoplasm (interpreted as fibroblasts) had consistent, moderate intracytoplasmic positive staining, while spindle cells with slender nuclei and thin scant cytoplasm (interpreted as fibrocytes) were either mildly positive or negative (Figure 3).

**Figure 4:**
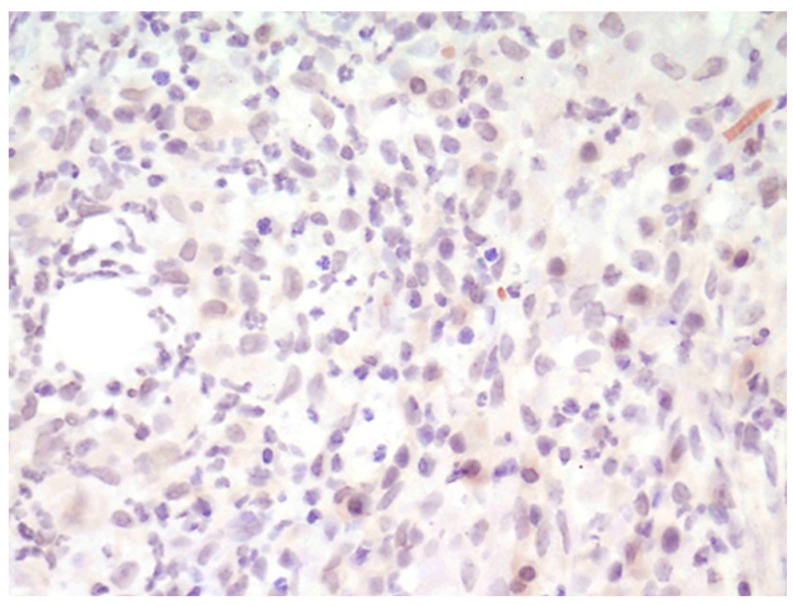
Injection-site granuloma. Macrophages and other inflammatory cells are negative for α_v_ integrin. IHC for α_v_ integrin, 400×.

In the injection-site granuloma, inflammatory cells (lymphocytes, plasma cells, and macrophages) were α_v_ integrin-negative (Figure 4), fibroblasts had moderate cytoplasmic positive labeling, and fibrocytes were inconstantly positive.

## 4. Discussion

In the study presented herein, we investigated the efficacy of the α_v_ integrin antibody in evaluating the surgical excision margins of FISS by assessing the differential IHC expression of this marker in neoplastic and non-neoplastic portions of surgical biopsies. This marker was selected since α_v_ integrin has previously shown promising results in optical guidance in surgery for spontaneous feline fibrosarcomas and tumors in other animal models [12,21]. Reactive fibroblasts and myofibroblasts, which exhibit morphological similarity to neoplastic cells, can represent an interpretative challenge for pathologists. From this perspective, we wondered whether α_v_ integrin could be proposed as an effective immunohistochemical marker specifically targeting neoplastic cells that could help with the histopathological evaluation of excision margins.

In the present study, however, while the anti-α_v_ integrin antibody showed good affinity in IHC for neoplastic cells, as demonstrated by the consistent positivity of spindle neoplastic cells in all FISS cases, immunohistochemical positive labeling was not limited to the neoplastic tissues. As a matter of fact, no difference in staining intensity was noted between neoplastic spindle cells and fibroblasts at the margins of the tumors, and fibrocyte labeling was inconstantly negative, not allowing a reliable and reproducible distinction between neoplastic, reactive, and quiescent spindle cells.

A mild discrepancy between intra-surgery fluorescent signals and immunohistochemical labeling in frozen tissue has been previously reported and attributed to the presence of perivascular neoplastic cells or the cross-reactivity of the antibody utilized with other integrins that are likely overexpressed in the inflamed peritumoral tissues [12]. However, no specific information was provided regarding the cell types that were labeled by α_v_ integrin. In our study, all tested tissues exhibited consistent negative staining for inflammatory cells, and positive α_v_ integrin staining was not limited to neoplastic cells. Instead, it was consistently observed in “reactive” fibroblasts, whether near or distant from inflammatory infiltration.

One hypothesis that could explain our results compared to those from the in vivo observations cited above [12] is the different reactivity of FFPE tissues to the IHC marker. Issues with the antigenicity of FFPE tissues have long been known and, not infrequently, represent an impediment when the expression of the same protein antigen is investigated in different types of matrices [22,23,24]. Another explanation could be that the anti-α_v_ integrin antibody used in the present study, which has been chosen because it is the only available antibody that has been proven effective in staining integrins in FFPE feline tissues [20], does not discriminate between all the integrins containing the alpha chain V (which, in mammals, include α_v_β_1_, α_v_β_3_, α_v_β_5_, α_v_β_6_, and α_v_β_8_) and, therefore, the signal could be expressed in a larger spectrum of cells. In future studies, it would be advantageous to obtain an appropriate antibody, currently unavailable, that is specific to the feline species, targets α_v_β_3_ integrin, and can be utilized in FFPE. The latter condition is imperative if the objective is to ascertain the potential utility of α_v_β_3_ integrin in assessing FISS margin status in routine diagnostic pathology.

Another important consideration is that the interpretation of fluorescent intra-surgical labeling is not an all-or-nothing approach, but a threshold was defined, under which value the signal was considered background. This background was considered by the authors to be doubtful or ‘‘inflammation-positive” [12], most likely related to the peripheral inflammatory infiltrates, consisting of lymphocytes and macrophages, that characterized feline fibrosarcoma. Since inflammation is known to precede the development of FISS and is implicated in its pathogenesis, the author concluded that “it is preferable to also remove this portion of the “doubtful” inflamed and weakly fluorescent peritumoral tissue, as it may potentially be irreversibly engaged in the transformation process”. Based on the observations in the present study, the “background” positivity seems not to be related to inflammatory cells, which were consistently negative for α_v_ integrin in all samples tested, but rather to connective tissue fibroblasts. In any case, the surgeon’s choice to remove this tissue is agreeable.

Although the primary hypothesis of our study was not verified, some interesting findings were observed. A marked and consistent difference in the staining of neoplastic cells was detected: while spindle cells in the fibrosarcoma subtype of FISS had a diffuse, mild-to-moderate intracytoplasmic positive staining, mononucleated round and multinucleated giant cells in pleomorphic sarcomas had a strong and very distinct membranous positivity. Conversely, multinucleated giant cells in osteosarcomas and macrophages in the granuloma and in panniculitis were either α_v_ integrin-negative or had a mild intracytoplasmic positive signal. Since both multinucleated giant cells and osteoclasts have been reported to be of monocyte/macrophage origin [19,25], a differential expression of α_v_ integrin could be specifically related to multinucleated giant neoplastic cells of pleomorphic sarcoma. Since the up-regulation of α_v_ integrin on the surface of neoplastic cells has been associated with the local invasion and metastatic dissemination of various cancers [26] and pleomorphic sarcoma has been reported to be the most aggressive subtype of FISS with a higher percentage of distant metastases compared with other histopathological subtypes [27], it can be suggested that α_v_ integrin plays a specific role in the FISS pleomorphic sarcoma variant.

## 5. Conclusions

We concluded that the IHC staining of FISS biopsies with the anti-α_v_ integrin antibody that we used is ineffective in distinguishing neoplastic tissue from non-neoplastic tissue, making it unsuitable for the histopathological evaluation of surgical excision margins. However, the clear membranous localization of α_v_ integrin in neoplastic round mononuclear and multinucleated giant cells of pleomorphic sarcomas is an interesting and novel finding, suggesting that α_v_ integrin may play an important role in the oncogenesis of the pleomorphic sarcoma variant of FISS. Further studies are warranted to elucidate the role of α_v_ integrin in feline pleomorphic sarcoma.

## Figures and Tables

**Table 1:** IHC results for α_v_ integrin.

Cases	Diagnosis	Main Lesion			Margins			
		Spindle	Mononucleated Round	Multinucleated Giant	Endothelial	Fibroblasts	Fibrocytes	Inflammatory Cells
1	Fibrosarcoma	++ (c)	\	\	+++	++ (c)	± (c)	-
2	Fibrosarcoma	++ (c)	\	\	+++	++ (c)	± (c)	-
3	Fibrosarcoma	++ (c)	\	\	+++	++ (c)	± (c)	-
4	Fibrosarcoma	++ (c)	\	\	+++	++ (c)	± (c)	-
5	Fibrosarcoma	++ (c)	\	\	+++	++ (c)	± (c)	-
6	Fibrosarcoma	++ (c)	\	\	+++	++ (c)	± (c)	-
7	Fibrosarcoma	++ (c)	\	\	+++	++ (c)	± (c)	-
8	Pleomorphic sarcoma	± (c)	+++ (m)	+++ (m)	+++	++ (c)	± (c)	-
9	Pleomorphic sarcoma	± (c)	+++ (m)	+++ (m)	+++	++ (c)	± (c)	-
10	Pleomorphic sarcoma	± (c)	++ (m)	+++ (m)	+++	++ (c)	± (c)	-
11	Osteosarcoma	+ (c)	+ (c)	+ (c)	+++	++ (c)	± (c)	-
12	Osteosarcoma	++ (c)	++ (c)	++ (c); ± (m)	+++	na	na	na
13	Osteosarcoma	+ (c)	+ (c)	+ (c)	+++	na	na	na
14	Osteosarcoma	++ (c)	++ (c)	++ (c); ± (m)	+++	na	na	na
15	Osteosarcoma	+ (c)	+ (c)	+ (c)	+++	na	na	na
16	Osteosarcoma	+ (c)	+ (c)	+ (c)	+++	na	na	na
17	Injection-site granuloma	\	\	\	+++	++ (c)	± (c)	-

−: no staining; +: mild positive staining; ++: moderate positive staining +++: intense positive staining; c, cytoplasmic; m, membranous; na, not available.

## Data Availability

The original contributions presented in the study are included in the article, further inquiries can be directed to the corresponding author.

## References

[1] Zabielska-Koczywąs K., Wojtalewicz A., Lechowski R. (2017). Current Knowledge on Feline Injection-Site Sarcoma Treatment. Acta Vet. Scand..

[2] Porcellato I., Menchetti L., Brachelente C., Sforna M., Reginato A., Lepri E., Mechelli L. (2017). Feline Injection-Site Sarcoma: Matrix Remodeling and Prognosis. Vet. Pathol..

[3] Martano M., Morello E., Buracco P. (2011). Feline Injection-Site Sarcoma: Past, Present and Future Perspectives. Vet. J..

[4] Ladlow J. (2013). Injection Site-Associated Sarcoma in the Cat: Treatment Recommendations and Results to Date. J. Feline Med. Surg..

[5] Hartmann K., Egberink H., Möstl K., Addie D.D., Belák S., Boucraut-Baralon C., Frymus T., Lloret A., Hofmann-Lehmann R., Marsilio F. (2023). Feline Injection-Site Sarcoma and Other Adverse Reactions to Vaccination in Cats. Viruses.

[6] Hartmann K., Day M.J., Thiry E., Lloret A., Frymus T., Addie D., Boucraut-Baralon C., Egberink H., Gruffydd-Jones T., Horzinek M.C. (2015). Feline Injection-Site Sarcoma: ABCD Guidelines on Prevention and Management. J. Feline Med. Surg..

[7] Giudice C., Stefanello D., Sala M., Cantatore M., Russo F., Romussi S., Travetti O., Di Giancamillo M., Grieco V. (2010). Feline Injection-Site Sarcoma: Recurrence, Tumor Grading and Surgical Margin Status Evaluated Using the Three-Dimensional Histological Technique. Vet. J..

[8] Carminato A., Vascellari M., Marchioro W., Melchiotti E., Mutinelli F. (2011). Microchip-associated Fibrosarcoma in a Cat. Vet. Dermatol..

[9] Martano M., Morello E., Iussich S., Buracco P. (2012). A Case of Feline Injection-Site Sarcoma at the Site of Cisplatin Injections. J. Feline Med. Surg..

[10] Carneiro C.S., de Queiroz G.F., Pinto A.C., Dagli M.L.Z., Matera J.M. (2019). Feline Injection Site Sarcoma: Immunohistochemical Characteristics. J. Feline Med. Surg..

[11] Couto S.S., Griffey S.M., Duarte P.C., Madewell B.R. (2002). Feline Vaccine-Associated Fibrosarcoma: Morphologic Distinctions. Vet. Pathol..

[12] Wenk C.H.F., Ponce F., Guillermet S., Tenaud C., Boturyn D., Dumy P., Watrelot-Virieux D., Carozzo C., Josserand V., Coll J.L. (2013). Near-Infrared Optical Guided Surgery of Highly Infiltrative Fibrosarcomas in Cats Using an Anti-Avß3 Integrin Molecular Probe. Cancer Lett..

[13] Gibbs-Strauss S.L., Rosenberg M., Clough B.L., Troyan S.L., Frangioni J.V. First-in-Human Clinical Trials of Imaging Devices: An Example from Optical Imaging. Proceedings of the 2009 Annual International Conference of the IEEE Engineering in Medicine and Biology Society.

[14] Grant D.S., Tashiro K.-I., Segui-Real B., Yamada Y., Martin G.R., Kleinman H.K. (1989). Two Different Laminin Domains Mediate the Differentiation of Human Endothelial Cells into Capillary-like Structures in Vitro. Cell.

[15] Suzuki S., Oldberg A., Hayman E.G., Pierschbacher M.D., Ruoslahti E. (1985). Complete Amino Acid Sequence of Human Vitronectin Deduced from CDNA. Similarity of Cell Attachment Sites in Vitronectin and Fibronectin. EMBO J..

[16] Pierschbacher M.D., Ruoslahti E. (1984). Cell Attachment Activity of Fibronectin Can Be Duplicated by Small Synthetic Fragments of the Molecule. Nature.

[17] Danhier F., Le Breton A., Préat V. (2012). RGD-Based Strategies to Target Alpha (v) Beta (3) Integrin in Cancer Therapy and Diagnosis. Mol. Pharm..

[18] Cossu J., Thoreau F., Boturyn D. (2023). Multimeric RGD-Based Strategies for Selective Drug Delivery to Tumor Tissues. Pharmaceutics.

[19] de Cecco B.S., Argenta F.F., Bianchi R.M., De Lorenzo C., Wronski J.G., Bandinelli M.B., da Costa F.V.A., Driemeier D., Pavarini S.P., Sonne L. (2021). Feline Giant-Cell Pleomorphic Sarcoma: Cytologic, Histologic and Immunohistochemical Characterization. J. Feline Med. Surg..

[20] Ahmed I., Mahmut S. (2021). Expression of Platelet Derived Growth Factor a, Its Receptor, and Integrin Subunit Alpha V in Feline Injection-Site Sarcomas. Acta Vet. Brno..

[21] Atallah I., Milet C., Henry M., Josserand V., Reyt E., Coll J., Hurbin A., Righini C.A. (2016). Near-infrared Fluorescence Imaging-guided Surgery Improves Recurrence-free Survival Rate in Novel Orthotopic Animal Model of Head and Neck Squamous Cell Carcinoma. Head Neck.

[22] Shi S.-R., Liu C., Taylor C.R. (2007). Standardization of Immunohistochemistry for Formalin-Fixed, Paraffin-Embedded Tissue Sections Based on the Antigen-Retrieval Technique: From Experiments to Hypothesis. J. Histochem. Cytochem..

[23] Hira V.V.V., de Jong A.L., Ferro K., Khurshed M., Molenaar R.J., Van Noorden C.J.F. (2019). Comparison of Different Methodologies and Cryostat versus Paraffin Sections for Chromogenic Immunohistochemistry. Acta Histochem..

[24] Mairaville C., Martineau P. (2021). Antibody Identification for Antigen Detection in Formalin-Fixed Paraffin-Embedded Tissue Using Phage Display and Naive Libraries. Antibodies.

[25] Helming L., Gordon S. (2009). Molecular Mediators of Macrophage Fusion. Trends Cell Biol..

[26] Liu F., Wu Q., Dong Z., Liu K. (2023). Integrins in Cancer: Emerging Mechanisms and Therapeutic Opportunities. Pharmacol. Ther..

[27] Dillon C.J., Mauldin G.N., Baer K.E. (2005). Outcome Following Surgical Removal of Nonvisceral Soft Tissue Sarcomas in Cats: 42 Cases (1992–2000). J. Am. Vet. Med. Assoc..

